# HO-1 and CD39: It Takes Two to Protect the Realm

**DOI:** 10.3389/fimmu.2019.01765

**Published:** 2019-07-26

**Authors:** Ghee Rye Lee, Shahzad Shaefi, Leo E. Otterbein

**Affiliations:** Departments of Surgery and Anesthesia, Beth Israel Deaconess Medical Center, Harvard Medical School, Boston, MA, United States

**Keywords:** heme, innate immunity, adenosine, metabolism, bioenergetics

## Abstract

Cellular protective mechanisms exist to ensure survival of the cells and are a fundamental feature of all cells that is necessary for adapting to changes in the environment. Indeed, evolution has ensured that each cell is equipped with multiple overlapping families of genes that safeguard against pathogens, injury, stress, and dysfunctional metabolic processes. Two of the better-known enzymatic systems, conserved through all species, include the heme oxygenases (HO-1/HO-2), and the ectonucleotidases (CD39/73). Each of these systems generates critical bioactive products that regulate the cellular response to a stressor. Absence of these molecules results in the cell being extremely predisposed to collapse and, in most cases, results in the death of the cell. Recent reports have begun to link these two metabolic pathways, and what were once exclusively stand-alone are now being found to be intimately interrelated and do so through their innate ability to generate bioactive products including adenosine, carbon monoxide, and bilirubin. These simple small molecules elicit profound cellular physiologic responses that impact a number of innate immune responses, and participate in the regulation of inflammation and tissue repair. Collectively these enzymes are linked not only because of the mitochondria being the source of their substrates, but perhaps more importantly, because of the impact of their products on specific cellular responses. This review will provide a synopsis of the current state of the field regarding how these systems are linked and how they are now being leveraged as therapeutic modalities in the clinic.

## Introduction

Metabolism requires complex relationships among substrates, the enzymes that catalyze their transformation and ultimately the products that are generated, to maintain cellular physiological functions. This is perhaps best illustrated by the intricacies of glycolysis and the Krebs cycle where glucose is ultimately converted to energy to fuel all cellular activities. Such pathways and cycles are intimately interrelated with others and in many instances cooperate to promote efficiency ultimately ensuring survival of the cell and organism. Heme and adenosine can be considered as cornerstones of the cell as well, fundamental components that sustain cellular homeostasis and contend with changes in the environment. Essential are the enzymes responsible for their generation and catabolism, which include the heme oxygenases (HO), and the ectonucleotidases (CD39/CD73). There is a relative paucity of evidence on the direct communication between HO and CD39. However, there are several groups that have studied the relationship between the products generated as a result of heme metabolism by HO, and those from the purinergic signaling pathway. Reports by us and others demonstrate that there is a clear relationship between these two systems and how they regulate the reaction of the cell to stress. Further, the products of their activity are potent bioactive molecules that in tandem regulate specific signaling events in the cell. If the enzymes, and therefore their products are absent, devastating outcomes result as the cell becomes highly susceptible to injury, and succumbs to death. In contrast, exogenous supplementation of each product results in powerful protection of the cellular and tissue realm.

## The Biology of Heme

### Synthesis

Heme, a ubiquitous molecular complex comprised of ferrous iron (Fe^2+^), and protoporphyrin IX, is found in all species in a transkingdom manner. In eukaryotes, it serves multiple physiological functions in support of cellular metabolism and survival when complexed as a hemoprotein. In hemoglobin and myoglobin, heme is critical for appropriate oxygen binding and delivery to remote site and without the heme contained within the hemoglobin tetramer, multicellular organisms would be unable to survive. As a part of mitochondrial cytochrome complexes, heme is responsible for transporting electrons that ultimately support aerobic respiration resulting in oxidative phosphorylation, and the generation of ATP. Heme is present in nitric oxide synthases, catalases, guanylate cyclases as well as mitochondrial oxidases and transcription factors such as Bach1 and RevErbα (1).

**Figure 1:**
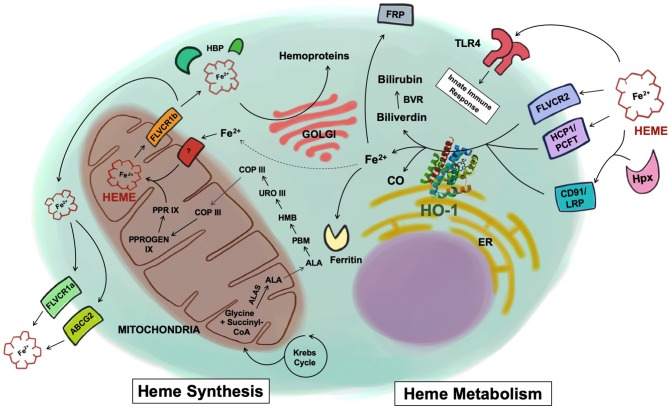
Heme synthesis and catabolism. Free heme is synthesized through a cascade of enzymatic reactions in the mitochondria and the cytosol. Once synthesized inside the mitochondria matrix, free heme is exported into the cytosol via the FLVCR1b transporter. Heme is then exported to the extracellular space via FLVCR1a or ABCG2, or chaperoned by various heme-binding proteins to be incorporated into hemoproteins within the cell. Heme uptake by the cell is mediated by a number of receptors including FLVCR2, HCP1/PCFT, or through the CD91/LRP receptor in complex with hemopexin. Heme has also been shown to be recognized by TLR4. Once inside the cells, heme is metabolized by HO-1 into biliverdin, Fe^2+^, and CO. Biliverdin is converted to bilirubin via biliverdin reductase. The Fe^2+^ is secreted through the exporter FRP, sequestered into ferritin, or recycled and utilized for heme synthesis (dotted line). ALAS, 5-aminolevulinic acid synthase; ALA, delta-aminolevulinic acid; PBM, porphobilinogen; HMB, 1-hydroxymethylbilane; URO III, uroporphyrinogen III; COP III, coproporphyrinogen III; PPROGEN IX, protoporphyrinogen IX; PPR IX, protoporphyrin IX; FLVCR, feline leukemia virus subgroup C receptor; ABCG2, ATP-binding cassette sub-family G member 2; HBP, heme binding protein; HCP1/PCFT, heme carrier protein 1/proton-coupled folate transporter; LRP, low-density lipoprotein receptor-related protein; Hpx, hemopexin; TLR4, toll-like receptor 4; HO-1, heme oxygenase-1; CO, carbon monoxide; BVR, biliverdin reductase; FRP, ferroportin; ER, endoplasmic reticulum.

Synthesis of heme is a highly conserved enzymatic process that takes place predominantly in erythroid cells of the bone marrow and hepatocytes. Beginning in the mitochondrial matrix, glycine and succinyl-CoA derived from the citric acid cycle are condensed to form delta-aminolevulinic acid (ALA) by the enzyme 5-aminolevulinic acid synthase (ALAS) (Figure 1). ALA synthase is a rate-limiting enzyme whose activity is negatively regulated by the levels of iron and heme present in the cell. Once ALA is formed, it is transported into the cytosol where it undergoes additional enzymatic processing, forming the intermediates porphobilinogen (PBM), 1-hydroxymethylbilane (HMB), uroporphyrinogen III (URO III), and coproporphyrinogen III (COP III), driven by the enzymes PBM synthase, PBM deaminase, URO III synthase, and URO III decarboxylase, respectively. COP III is then transported back into the mitochondrial matrix, where it is converted into protoporphyrinogen IX, catalyzed by COP oxidase, and then into protoporphyrin IX by protoporphyrinogen oxidase (2). Subsequently, Fe^2+^, which is acquired from the diet or recycled from senescent erythrocytes that have been engulfed by macrophages, a process known as erythrophagocytosis, is inserted into protoporphyrin IX by the enzyme ferrochelatase forming the final heme tetrapyrol configuration (Figure 1) (3, 4). Defects in this tightly concerted process of heme synthesis can result in a series of clinical pathophysiological phenotypes including anemia, defective erythropoiesis, and porphyrias (5).

Once the heme molecule is synthesized inside the mitochondrial matrix, it must be transported to other cellular compartments to support different physiological functions. For instance, heme needs to reach the mitochondrial intermembrane space to bind to cytochrome C or the mitochondrial intermembrane to bind to cytochrome complex III or IV so that these enzymes can properly perform mitochondrial respiration (6). In order to be combined with cytosolic proteins such as globins, nitric oxide synthases, and guanylyl cyclases, heme is exported into the cytosol from the mitochondrial matrix, most likely via the transporter feline leukemia virus subgroup C receptor (FLVCR) 1b (7, 8).

Heme also plays a critical role in gene regulation. In the nucleus, heme inhibits the activity of the transcription repressor Bach1 through direct binding (9) or participates in microRNA processing by binding to the RNA-binding protein DiGeorge critical region-8 (10). Additionally, heme is delivered to other organelles within the cell to be incorporated as a hemoprotein for other purposes. For instance, heme is required in peroxisomes to form catalase, an antioxidant enzyme that is actively made and secreted by hepatocytes and erythrocytes (11). In neutrophils and to a lesser degree in monocytes, heme is transported to azurophilic granules to form myeloperoxidase, a peroxidase that is secreted in response to microbial challenges (12). Besides the newly synthesized heme in the mitochondria, cells can obtain heme from the extracellular space through the heme carrier protein-1/proton-coupled folate transporter (HCP1/PCFT), or FLVCR2 (13, 14). To ensure proper delivery to target hemoproteins and organelles within the cell, heme is thought to be chaperoned by various cytosolic heme-binding proteins (HBPs), such as glutathione S-transferase (15), liver fatty acid binding protein 1 (16), heme-binding protein 23 (17), and p22 HBP (18). More investigations are needed to fully elucidate the exact mechanism by which heme molecules are moved within the cell and incorporated into hemoproteins.

### Elimination and Catabolism

When not bound to hemoproteins, intracellular free heme can be detected under physiological conditions at concentrations of 100 nM (19). However, when levels exceed this amount as a result of cellular injury or due to lack of clearance, it becomes dangerous mainly due to the ability of the otherwise caged iron atom, facilitating generation of toxic oxygen free radicals via Fenton chemistry. Here ferrous iron reacts with hydrogen peroxide to generate the highly toxic hydroxyl radical. Additionally, due to its lipophilic nature, heme can readily intercalate into cell membranes, resulting in lipid peroxidation, destabilization of the cell membrane, and eventually cell rupture, making it a powerful hemolytic, and cytolytic agent. Heme is also recognized by the Toll-4 receptor (TLR4) acting as a Damage-Associated Molecular Pattern (DAMP) to activate leukocytes and trigger pro-inflammatory cytokine secretion (1, 20). Heme has also been shown to increase expression of adhesion molecules including ICAM-1, VCAM-1, E-selection, and P-selectin resulting in endothelial cell activation that leads to leukocyte mobilization, and recruitment (21, 22). In addition to the Fenton reaction, heme can promote formation of reactive oxygen species (ROS) through enzymatic reactions that involve NADPH oxidase or through non-enzymatic reactions by converting hydroperoxides into toxic free radicals that can cause cell and tissue damage (23). These cytotoxic properties of free heme point to its role in the pathogenesis of many immune-mediated inflammatory diseases such as sickle cell anemia, malaria, hemorrhage, ischemia reperfusion injury, and infection (24).

To protect cells and tissues from the aforementioned toxicity of free heme, its levels are tightly regulated systemically. To prevent the free heme from accumulating within the cell, it can be exported via FLVCR or ABCG2 (ATP-binding cassette subfamily G member 2) (25, 26). A surplus amount of extracellular free heme is principally eliminated from the serum through the heme-binding protein hemopexin, which recognizes and internalizes free heme by the CD91/LRP1 scavenger receptor, but also through its ability to bind to haptoglobin, and albumin (27, 28). The internalization of the heme-hemopexin complex and the subsequent recycling of heme is performed by hepatocytes and macrophages of the liver and the spleen (27, 29). Liver is thought to be the primary site that clears the heme-hemopexin complex but other tissues such as the human placenta and the brain are also reported to clear it (30, 31). The high expression of CD91/LRP1 in the placenta during pregnancy and the activity of the heme-hemopexin clearance system in the brain after subarachnoid hemorrhage suggest that various forms of stress, where there are changes in vascular permeability, organs other than the liver may see the heme-hemopexin complex entering tissue parenchyma. Therein, it can be internalized and processed by resident macrophages and other cell types (32, 33). After internalization, intracellular free heme is principally metabolized by HO and every cell contains one or both isoforms of heme oxygenase. HO-1 is the inducible form regulated in large part by the transcription factors Nrf2 and Bach1 while HO-2 is constitutive and primarily found in the brain, testes and endothelium where it contributes to vasomotor tone and oxygen sensing (34–36). Within the cell, heme can be rapidly degraded into iron, biliverdin, and carbon monoxide (CO). Iron is sequentially sequestered into ferritin and biliverdin is further converted to bilirubin by biliverdin reductase (Figure 1) (37).

## Heme Oxygenase-1 and Cellular Protection

In addition to its primary role in heme metabolism, HO-1 is well-known as a “master” stress-response gene due to its central role in the host's response to changes in the environment, where it serves to preserve homeostatic maintenance of cells and tissues under pathophysiological conditions. In addition to heme, other stimuli induce HO-1, such as pathogens (38), tissue damage (37, 39), hypoxia (40), hyperoxia (41), inflammatory cytokines (42), ultraviolet light (43), and oxidants (44). The effects of HO-1 have been cemented as being cytoprotective, especially under inflammatory conditions. There are well-documented studies showing that the induction of HO-1 leads to remarkably better survival in numerous *in vivo* models of tissue injury and infection, while the lack of HO-1 is highly detrimental (45–48). HO-1 deficient individuals exhibit heightened susceptibility to stress and increased inflammatory indices such as leukocytosis and thrombocytosis with a significantly shortened life span (49–51). HO-1 deficient mice mimic the human phenotype with increased sensitivity to a plethora of stressors (52). Increasing data have clearly solidified that the mechanism of HO-1 cytoprotection is mediated through the generation of one or more of its products, including the bile pigments, biliverdin/bilirubin, and CO, and likely through the removal of prooxidant iron. Each of these bioactive products has been well-studied and functions through various signaling pathways depending on the cell type and model system being studied.

### Protective Products

The bile pigments are powerful antioxidants as well as signaling molecules that regulate inflammation, cell survival and innate immune responses. Exogenous administration of the green pigment biliverdin is protective against diverse pathophysiological conditions *in vivo* including endotoxin-induced lung injury (53), colitis (54), cecal ligation and puncture-induced sepsis (55), corneal epithelial injury (56, 57), hepatic ischemia-reperfusion injury (IRI) (58), and intestinal transplantation (59). In these models, biliverdin improved survival and ameliorated disease progression and complications by attenuating pro-inflammatory cascades, such as leukocyte infiltration, secretion of pro-inflammatory cytokines IL-6, IL-1β, and TNF, and reducing the oxidative burden, while simultaneously enhancing anti-inflammatory responses such as secretion of IL-10 and promoting tissue repair. Biliverdin has also been shown to confer protection by mediating T-cell responses (60). In a model of cardiac transplantation in rats, exogenous biliverdin promoted tolerance to cardiac allografts by halting T cell proliferation, inhibiting activation of nuclear factor of activated T-cells (NFAT), and the secretion of interferon-gamma (IFN-γ) by T helper type 1 (Th1) cells (60). Additionally, biliverdin can interfere with the complement system, specifically at the C1 step in the classical pathway, and its anti-complement role in the prevention of anaphylaxis has been demonstrated (61). Biliverdin also possesses antiviral property (62, 63), inhibiting hepatitis C viral replication and the activity of non-structural 3/4A protease (63). Administration of biliverdin ameliorated vascular injury and the formation of intimal hyperplasia by reducing endothelial cell apoptosis, and vascular smooth muscle cell migration (64). While these benefits can be attributed to biliverdin's antioxidant properties, they are actually supported by data sets that show that biliverdin reductase is an important contributor to cell function and survival. In macrophages, binding of biliverdin to biliverdin reductase activates PI3K-Akt signaling cascade that leads to the secretion of the anti-inflammatory cytokine, IL-10 (65). In the animal model of acute liver injury, binding to biliverdin reductase is required for biliverdin to block TLR4 expression and in part regulate macrophage chemotaxis to C5a (66, 67).

The salutary effects of biliverdin and biliverdin reductase may also be mediated in part by the generation of bilirubin. While an extremely high level of bilirubin is neurotoxic, a moderately increased level of serum bilirubin has been shown in numerous reports to be associated with reduced risk for cardiovascular disease and diabetes (68–74). This potential protective role of bilirubin can in part be explained by its potent anti-oxidant properties (75). Bilirubin can efficiently trap hydroperoxyl radicals and protect lipids from peroxidation (76). Bilirubin is anti-inflammatory and immunosuppressive and can inhibit adhesion of neutrophils to endothelium by blocking TNF-induced upregulation of E-selectin, VCAM-1, and ICAM-1 through inhibiting nuclear translocation of NF-κB (77, 78). In an animal model of endotoxin challenge, bilirubin blocks lipopolysaccharide (LPS)-induced tissue damage by blocking the expression of inducible nitric oxide synthase (iNOS), and thus the production of nitric oxide (79). Like biliverdin, bilirubin also modulates T cell responses to exert immunosuppressive effects. In an experimental model of islet transplantation, exogenous bilirubin stimulated expansion of Foxp3^+^ regulatory T cells (Tregs) at the site of the islet allografts, improving the function of the transplanted allograft (80, 81). In contrast, bilirubin induces apoptosis of reactive CD4^+^ T cells, downregulating MHCII and T-cell receptor (TCR) signaling pathways (82). In T helper type 17 (Th17) cells, ligation of the aryl hydrocarbon receptor to bilirubin results in abrogation of inflammatory bowel disease (83). The salutary effects of bilirubin are corroborated in additional preclinical animal models, such as vascular injury (84), hyperoxia (85), organ transplantation (81, 86, 87), and autoimmune encephalomyelitis (82), making bilirubin a potential therapeutic target in diseases characterized by oxidative stress and hyper-immune responses.

CO is a potent gasotransmitter with multiple functionalities including regulation of cell survival and proliferation, and innate immunity. As noted above, CO binds principally to hemoproteins such as guanylate cyclase, nitric oxide synthase, and the mitochondrial oxidases where it modulates their activity either positively or negatively. In preclinical models, exogenous CO provides potent salutary effects against organ transplantation, ischemia reperfusion injury, shock (endotoxin/infection/hemorrhagic), sepsis, vascular injury, malaria, and acute lung or liver injury (46, 88, 89). Of the three products generated during heme metabolism, CO has been the most extensively studied in animal models and it is the only one whose protective effect has been evaluated as a potential therapeutic agent in clinical trials to treat lung fibrosis (NCT01214187), sickle cell disease (NCT02411708, NCT02672540), and to reduce rejection of a transplanted organ (NCT00531856, NCT02490202). Currently, there are ongoing clinical trials that are testing the safety and the efficacy of CO to treat acute respiratory distress syndrome (NCT03799874, NCT02425579). In addition, the safety of an orally available formulation of CO is being tested (NCT03926819).

CO can exert protective effects by being pro- or anti-apoptotic, pro- or anti- proliferative, and pro- or anti-inflammatory depending on the target cells and the host's pathophysiological status. In T cells, CO exposure leads to Fas/CD95-induced apoptosis by activating caspase-8,-9, and−3 and upregulating the pro-apoptotic protein FADD while down-regulating the anti-apoptotic protein Bcl-2 (90). In contrast, CO protects endothelial cells from apoptosis by activating the p38 mitogen-activated protein kinase (MAPK) signaling pathway (91). In the presence of pathogens, CO can exert pro-inflammatory effects on macrophages by boosting their capacity to phagocytose by upregulating TLR4 (92), and the NLRP3 inflammasome (38). On the other hand, when administered prior to an inflammatory insult, CO imparts anti-inflammatory effects on macrophages by inhibiting TNF and IL-1β secretion and promoting production of the anti-inflammatory cytokine IL-10 via the activation of MAPK signaling (93). CO is both pro- and anti-proliferative in the vascular compartment. In models of vascular stenosis, CO prevents smooth muscle cell growth, and in scenarios where there has been endothelial cell denudation such as after balloon angioplasty, CO promotes endothelial cell proliferation (94, 95). Pulmonary hypertension results in part via dysregulated proliferation of the pulmonary artery smooth muscle cells. CO treatment, when initiated at the peak of stenosis and right heart hypertrophy, elicits a pro-apoptotic response to eliminate the smooth muscle cell mass, in part by increasing the generation of endothelium-derived nitric oxide production (96).

Iron, another byproduct of HO-1, plays a critical role in fundamental physiological functions. As mentioned above, iron is a central component of the heme structure, and therefore is required for the wide range of biological activities that hemoproteins perform including mitochondrial function, respiration, oxygen delivery, and storage. Iron is also needed as a cofactor in iron-sulfur cluster proteins, such as DNA polymerases, DNA helicases, and DNA primases, in order to carry out DNA replication and repair (97). Moreover, iron is utilized by oxo di-iron (Fe-O-Fe)-containing enzymes, such as ribonucleotide reductase, to support DNA synthesis (98). Iron is acquired through the diet absorbed by intestinal cells, but the majority is recycled from senescent red blood cells by splenic macrophages as heme is degraded (98).

An imbalance in the level of iron available for cells can be detrimental to the host. Insufficient amount of iron prevents the cell from performing basic functions that support life, especially erythropoiesis and can result in anemia. Likewise, iron overload is damaging to cells due to its ability to rapidly initiate the Fenton reaction with hydrogen peroxide, generating toxic ROS. Due to this reactive nature, iron in excess is implicated in chronic inflammation, cardiovascular, and neurodegenerative diseases (99). To maintain iron homeostasis, cells are continuously fine-tuning the intracellular levels either by exporting free iron through the exporter ferroportin or sequestering it into a redox-inactive form that occurs in the cytosol by ferritin (100).

As heme is metabolized by HO-1, free iron is released, increasing the expression of ferroportin and ferritin, showing a close link between the iron-clearance system, and the activity of HO-1 (101, 102). Although the ferrous iron itself is a harmful byproduct of heme metabolism, it is the subsequent expression of ferroportin, and ferritin that contributes to the protective effect of HO-1. In fact, it has been shown that the cytoprotective effect of HO-1 is dependent on the expression of ferritin. For example, in a model of cisplatin-induced nephrotoxicity, the loss of ferritin led to aggravation of acute kidney injury, despite high expression of HO-1 that is otherwise renoprotective (103, 104). Further, the role of ferritin in mediating the protective effect of HO-1 against oxidative stress is corroborated in lupus nephritis (105). Collectively, these reports demonstrate that the protective effects of HO-1 requires ferritin to chelate the iron.

Heme turnover is a key component of cell survival both as an active component of numerous proteins, but also as it is metabolically deconstructed by HO-1 into powerful products with effective signaling mechanisms that regulate important cellular functions.

## The Biology of Adenosine Triphosphate

Adenosine 5′-triphosphate (ATP) is indispensable for continued cellular metabolism and survival of the organism. As the universal “energy currency,” ATP is required to sustain the majority, if not all of physiological processes ongoing in living organisms. As this topic is the theme of many of the reviews in this series, we touch upon the biochemistry and biology minimally here, and refer the reader to these other reports for more detail. Basic physiological processes such as muscle contraction, synthesis of macromolecules, active transport, and thermogenesis require energy from ATP. As with heme, ATP is generated primarily in the mitochondria where its generation relies strictly on cytochrome c oxidase, a transmembrane hemoprotein complex that serves as the terminal enzyme in the respiratory electron transport chain and is regarded as one of the major sites for oxidative phosphorylation.

Despite its pivotal role in cellular metabolism, free ATP in excess can be toxic to the host and lead to cell death resulting from protracted stimulation of P2X_7_ receptors (106). Intracellular ATP can be released into the extracellular space under a variety of stress conditions such as autophagy, inflammation, hypoxia or apoptosis. Similar to heme, ATP is considered as a DAMP where it can act to signal to adjacent or remote cells via specific sets of receptors. ATP is a strong chemoattractant for phagocytes such as neutrophils and monocytes in a P2Y_2_-receptor-dependent manner (107, 108). In dendritic cells (DCs), ATP can increase their mobility through activation of P2X_7_ receptors as well as pannexin 1 channels and in the setting of allergen-driven lung inflammation, ATP has been shown to intensify T helper type 2 (Th2) cell responses by activating and recruiting DCs (109, 110). In the intestine, extracellular ATP can induce the release of pro-inflammatory cytokines and chemokines from mast cells through a P2X_7_-dependent manner, exacerbating intestinal inflammation (111). Additionally, in kidney injury, the loss of P2X_7_ remarkably improved the disease progression of unilateral ureteral obstruction, as evidenced by a significant decrease in inflammation, apoptosis in epithelial cells, and renal fibrosis (112). These studies collectively show the detrimental effect of the overstimulation of the purinergic receptor P2X_7_, with ATP as the potent ligand. On the contrary, an excess amount of ATP can confer immunosuppression by inhibiting proliferation and inducing cell death in activated CD4^+^ T cells and enhancing proliferation of Tregs (113). This contrasting response of different subsets of T cells to high levels of ATP is most likely adapted in order to dampen and resolve the hyper-inflammatory conditions where cellular, and tissue necrosis leads to ATP secretion. Immune cells are not the only cell type that is affected by ATP. It has been shown that ATP promotes vasodilation by activating the purinergic P2Y_2_ receptors on endothelial cells to promote their release of prostacyclin and nitric oxide (114–116). In the central and peripheral nervous system, ATP functions as a neurotransmitter (117–119) and in part regulates sleep regulation and memory formation (120, 121).

One example of a unique functional role for extracellular ATP is host-pathogen recognition. We have shown that extracellular ATP, by binding to P2X_7_ purinergic receptors on macrophages induces an inflammatory by activating the NLRP3 inflammasome that leads to subsequent caspase-1-dependent processing and secretion of pro-inflammatory cytokine IL-1β and IL-18. Indeed, macrophage-derived HO-1/CO compels bacteria to generate large amounts of ATP that is then recognized by the host as a danger signal and leads to enhanced activation of the NLRP3-Caspase-1 pathway and effective bacterial recognition and killing (122). This fundamental biological process by which host cells recognize pathogens, which is likely ancient in design, authenticates the intricate relationship between heme, ATP and host cell physiology.

## Regulation of ATP Levels: Role of CD39/CD73

**Figure 2:**
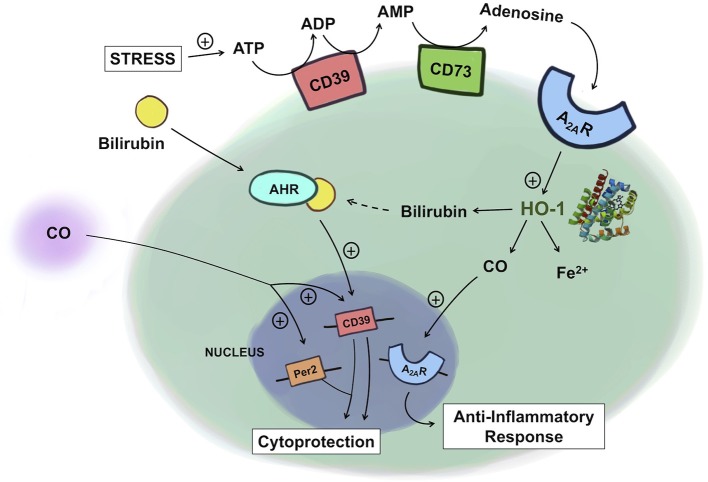
Crosstalk between heme and purine metabolism. Under conditions of stress, ATP levels rise and ATP is subsequently degraded into ADP and AMP by CD39 and then into adenosine by CD73. Adenosine binding to A_2A_R leads to upregulation of HO-1, which increases the production of CO. CO in turn can further amplify A_2A_R expression, leading to an enhanced anti-inflammatory response in macrophages. Exogenous administration of CO induces the expression of CD39 and Per2, conferring cytoprotection against kidney ischemia/reperfusion injury. In Th17 cells, bilirubin binds to the AHR, which together upregulates CD39, providing immunosuppressive, protective effects in experimental colitis. ATP, adenosine triphosphate; ADP, adenosine diphosphate; AMP, adenosine monophosphate; A_2A_R, adenosine A_2A_ receptor; AHR, aryl hydrocarbon receptor; HO-1, heme oxygenase-1; CO, carbon monoxide.

Under physiological conditions, ATP is continuously secreted into the extracellular space through active channel transport, or packaged in vesicles (106). However, under perturbed states, in order to prevent the cytotoxic and excessive pro-inflammatory effects of ATP, its extracellular level is controlled by the purinergic system involving two ectonucleotidases. 5′Ecto-nucleoside triphosphate diphosphohydrolase (CD39) converts ATP and ADP less efficiently to AMP. Ecto-5′nucleotidase (CD73) further degrades AMP into adenosine (Figure 2). CD39 is constitutively expressed in spleen, placenta, thymus, lung, and various cell types such as endothelial cells, NK cells, monocytes, lymphocytes, and Tregs. CD73 is principally expressed in colon, brain, kidney, lung and heart as well as on endothelial cells and Tregs (123). ADP, AMP, and adenosine are all bioactive molecules that participate in diverse cellular functions. Adenosine, generated by dephosphorylation of the adenine nucleotides in response to stress regulates numerous functions in the cell, but is perhaps been described as an inhibitor of inflammatory responses of neutrophils and monocytes/macrophages (124).

## CD39/CD73 in Inflammation and Immunity

Upregulation of CD39 and CD73 is observed in response to various instances of tissue damage and inflammation and server to degrade ATP to prevent the toxic and pro-inflammatory effects of ATP and promote the formation of adenosine, a potent anti-inflammatory molecule. Adenosine induces a series of anti-inflammatory responses through various immune cells by interacting with one or more G-protein coupled surface adenosine receptors, A_1_, A_2A_, A_2B_, and A_3_. For example, adenosine reduces leukocyte recruitment and adhesion to the endothelium, as well as phagocytosis and ROS production by neutrophils (124). Adenosine also inhibits M1 macrophage-mediated pro-inflammatory responses, such as TNF and IL-6 secretion, predominantly through the A_2A_ receptor. Moreover, adenosine is a potent vasodilator at the site of injury, which is thought to contribute, in part to reduced swelling (125). Given the opposing effects of ATP and adenosine that exist during an inflammatory response, the CD39/CD73 axis plays a central role in mediating pro- and anti-inflammatory responses that are tightly regulated to ensure cellular defense and effective resolution.

## Relationship Between Heme and ATP

Heme degradation and ATP metabolism mediated by HO-1, and CD39/CD73, respectively, are essential physiological processes required for cellular function and survival. As detailed above, each enzymatic system generates products that impact cellular function. Accumulating evidence in these two fields has unveiled their substantial role in regulating immune responses to tissue damage and inflammation. However, until recently no studies have shown an interaction between HO-1, CD39/CD73, and their products. What is emerging is a clear crosstalk between the mediators of HO-1 metabolism and purinergic signaling.

Recent works by Haschemi et al. and Weigel et al. are beginning to demonstrate an intricate physiologic link between heme and purinergic signaling in inflammation (38, 126). Haschemi et al. showed a positive feedback loop among HO-1, CO, and adenosine receptors by which they collectively impart immunomodulatory effects. Adenosine increases HO-1 in macrophages, which via CO, induces A_2A_ receptor expression, and leads to inhibition of LPS-induced TNF production. Absent the A_2A_ receptors, such immunomodulatory effects of HO-1, and CO were abolished. This finding is in line with the findings from the murine model of acute pulmonary inflammation where the effects of HO-1 are mediated through the adenosine receptors A_2A_, and A_2B_. Without these receptors, HO-1 induction failed to reduce the LPS-induced production of chemokines, neutrophil infiltration into the airway, and changes in the pulmonary vascular permeability (127).

When initiated after a live bacterial infection, treatment with CO enhances pro-inflammatory responses in macrophages and more effective bacterial killing. In the presence of CO, either endogenously generated, or administered exogenously compel bacteria to generate more ATP. The ATP production in turn binds to purinergic P2X_7_ receptors on macrophages, resulting in activation of the NALP3 inflammasome. In this paradigm the bacterial-derived ATP caused by the presence of CO is not broken down into adenosine by CD39/73, at least not initially, but is used to enhance macrophages' pro-inflammatory capabilities. Activation of the NALP3/Caspase1 leads to increase processing of pro-IL-1β into active IL-1β that is secreted, and contributes to the bacterial killing. Whether the bactericidal effects are directly due to IL-1β or indirectly through an auto-activation mechanism remains unclear. The model that was elucidated involved a 2-hit system akin to that described in T cell activation. Such a system prevents unnecessary activation of leukocytes. Endotoxin increases HO-1 expression and the generation of CO and is considered as the first signal. If a live bacterium is present, CO will drive the increase in ATP vis-à-vis its respiratory complexes, that then acts on the macrophage P2X_7_ receptor which is signal two. Such a system is energy efficient. Were only one signal required, host macrophages would likely exist in a constant unfettered over-stimulated state, particularly in the peritoneum where intestinal endotoxin levels fluctuate with intestinal microbes in close proximity. In such instances, mounting a full inflammatory response would be metabolically costly, inefficient, and potentially detrimental to the organism. A similar process may occur in neutrophils and involves increased phagocytosis.

These two reports not only illustrate the elaborate connection between these two seemingly independent pathways but also depict how they can exert different immune responses that are most favorable to host survival in a context-dependent manner by interacting with different components of the signaling pathway.

Products of HO-1 metabolism, specifically bilirubin, as well as CD39/73 have been implicated as potential therapeutic targets for inflammatory bowel disease (IBD). In the human gastrointestinal tract, CD39 is expressed on various immune cell types and endothelial cells and CD73 is predominantly expressed on the apical surface of intestinal epithelial cells (128). HO-1 is expressed in intestinal epithelial cells, endothelial cells and mononuclear cells (129). In various preclinical models of IBD, lack of either CD39/73, or HO-1 increases disease severity (130–133). This effect is in part explained by the accumulation of ATP due to lack of CD39/73. The loss of protection observed with HO-1 deficiency is in part due to the lack of product availability including biliverdin/bilirubin, and CO. Exogenous administration of CO, biliverdin, or bilirubin was protective against IBD. In the murine model of dextran sodium sulfate (DSS)-induced colitis, bilirubin prevented migration of leukocytes and eosinophils to the intestine via VCAM-1-mediated signaling (134). Longhi et al. reported that the mechanism of bilirubin's salutary action in IBD involves CD39 on Th17 cells (83). In IBD patients, the reduced ratio of Tregs to Th17 cells and thus higher pro-inflammatory activities of Th17 cells are thought to be important in the induction and persistence of the disease (135, 136). The mechanism of bilirubin-induced protection is in part related to upregulation of CD39 with an increase in the frequency of CD39^+^ Th17 cells in the experimental colitis model. Further, this upregulation of CD39 is mediated by the aryl hydrocarbon receptor (AHR) because bilirubin, as a natural ligand for AHR, activates AHR to upregulate CD39 expression in Th17 cells. Importantly, Th17 cells isolated from IBD patients were unresponsive to the immunomodulatory effects of bilirubin due in part to defective AHR expression. This report features how a product of heme metabolism regulates the expression of CD39 expression to influence the pathogenesis of IBD. The examples described above begin to elucidate and support the relationship between these two cytoprotective gene systems (Figure 2).

The protective role of HO-1 and the purinergic signaling pathway in renal IRI has been well-documented. Exogenous CO at low concentrations administered by inhalation, or delivery using a CO-releasing molecule or as a CO-saturated solution has become an exciting therapeutic intervention to prevent renal IRI (137). The mechanism of protection afforded by CO involves its ability to inhibit apoptosis of endothelial cells and the infiltration of immune cells through the downregulation of adhesion molecules and enhanced presence of tolerogenic Foxp3^+^ Tregs. Recently, Correa-Costa et al. demonstrated that CO protects the kidney from IRI by modulating purinergic signaling (137). Specifically, CO conferred protection by increasing serum levels of erythropoietin and the expression of the circadian rhythm protein Per2 via upregulation of CD39 and the A_2B_ receptor in the kidneys. In CD39 knockout mice, the protective effect of CO was lost. In addition, the pharmacologic blockade of A_2_ receptors reversed CO-induced renal protection. These are the first data sets that show that purinergic mediators are required for CO to confer protection against renal IRI, again highlighting the tight link between heme metabolism and purinergic signaling (Figure 2).

Over the last decade, our laboratory and others have begun to elucidate the interrelationship between heme and purinergic metabolism in preclinical models in inflammatory states in all tissues and central to appropriate responses to pathologic states such as sepsis, acute lung injury, IBD, and IRI. Our increased understanding in this field will identify and foster new therapeutic target and even devise new therapeutic interventions. Indeed, CO is in multiple clinical trials as is modulation of purinergic signaling. In summary, we have described in this review a summary of two elementary, but essential metabolic enzyme systems that act in large part through the generation of their products to regulate cellular responses to stress. We would argue that they are homeo “dynamic” in that they serve in a manner that is optimal for survival and not necessarily to restore the cell to an arbitrary basal homeo “static” state. Indeed, unless the environment becomes static, the organism is constantly in flux and adapting as necessary. As such, genes such as the heme oxygenases and the ectonucleotidases subserve this underlying need to adjust to befit the need of the tissue. Furthering our understanding of how they interact will not only provide novel discoveries toward understanding disease pathogenesis, but more importantly contribute to the design of potential therapies to protect the corporeal realm against inflammatory disease.

## Author Contributions

GL, SS, and LO contributed equally to the content. GL created the figures.

### Conflict of Interest Statement

LO is a scientific consultant for Hillhurst Biopharma. The remaining authors declare that the research was conducted in the absence of any commercial or financial relationships that could be construed as a potential conflict of interest.
